# Long-Term Sustainability of Timely Emergency Department Analgesia for Fractures: A Time Series Study

**DOI:** 10.1097/pq9.0000000000000026

**Published:** 2017-06-02

**Authors:** Emily C. Sterrett, Eileen Murtagh Kurowski, Terri L. Byczkowski

**Affiliations:** From the Department of Pediatrics, Division of Pediatric Emergency Medicine, Cincinnati Children’s Hospital Medical Center, Cincinnati, Ohio.

## Abstract

**Objectives:**

To determine the long-term sustainability and unintended consequences of a quality improvement project to improve the timeliness of intravenous (IV) opioid administration to patients with long-bone extremity fractures within a dynamic pediatric emergency department.

**Methods:**

A retrospective study of patients with long-bone extremity fractures was conducted using electronic medical record data from 2007 to 2014. The primary outcome was the percentage of patients receiving timely IV opioids. Control charts and time series models were used to determine if changes in the clinical microenvironment were associated with shifts in the outcome measure. Unintended consequences included patients receiving potentially avoidable IVs and use of the quality improvement process for patients without long-bone extremity fractures.

**Results:**

Improved timeliness of IV opioids was sustained. The type of physician who staffed the process and optimization of faculty staffing hours were associated with a 9.6% decrease and 11.8% increase in timely IV opioids, respectively. Implementation of the IV opioid process was not associated with increased placement of potentially avoidable IVs. Of patients receiving the IV opioid process, 22% did not have a long-bone extremity fracture, of whom 91% were diagnosed with a different painful injury.

**Conclusion:**

Sustainability of IV opioid timeliness was robust, despite changes in the clinical microenvironment. Changes in physician staffing and responsibilities in a pediatric emergency department may be especially important to consider when planning future improvement initiatives. Our findings support the importance of higher reliability interventions, such as identification and utilization of existing patterns of behavior, as high yield for sustaining outcomes.

## BACKGROUND

The keys to long-term sustainability of improved outcomes resulting from quality improvement (QI) work are not well understood as the majority of QI publications track measures for only 1 year following the initial work.^1,2^ Also, there is a paucity of literature on unintended consequences resulting from QI work.^3–9^

To address these gaps, we conducted a retrospective study of a QI project to improve the timeliness of intravenous (IV) pain medication for patients with clinically apparent long-bone extremity fractures in our pediatric emergency department (PED).^10^ The resulting process, implemented in October 2007, continues to be our standard of care. Changes in our clinical microenvironment since 2007 provided an ideal opportunity to measure the sustainability of the original outcome and examine potential unintended consequences resulting from implementing and sustaining this process. Studies such as this are a first step to exploring and understanding the robustness of improvement work within an ever-changing clinical environment.

### Process for Timely Pain Medication

**Fig. 1.**
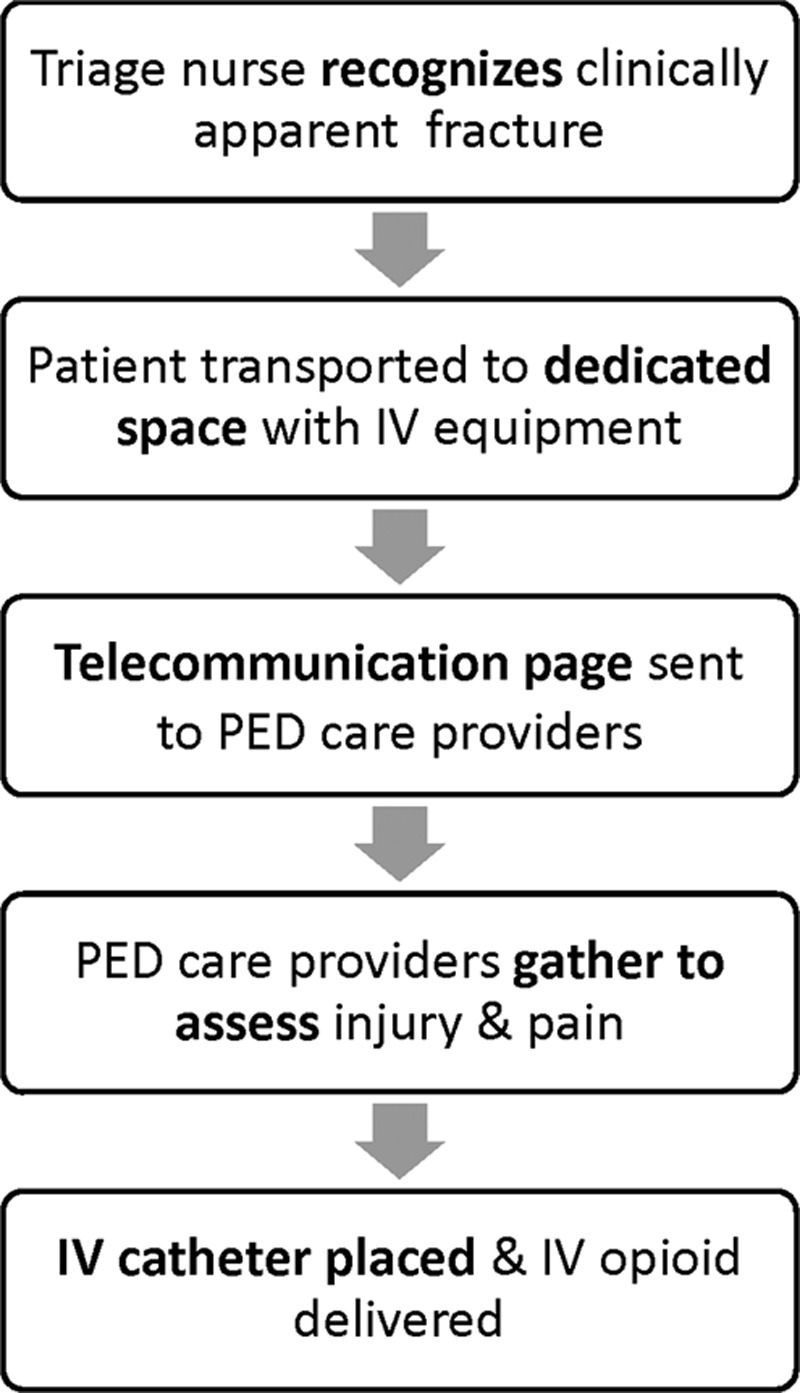
Process map for IV opioid process.

In 2007, Iyer et al.^10^ used the Model for Improvement^11^ to improve the timeliness of IV opioids for patients with clinically apparent long-bone extremity fractures. The IV opioid process, as it exists today, was implemented in October 2007 (Fig. 1). The primary outcome measure was the proportion of patients with a long-bone extremity fracture who received their first dose of IV opioid within 45 minutes of arrival. Special cause variation indicated improvement in this outcome measure within 60 days of implementation. The results were monitored for an additional 20 months showing that the initial gains were sustained. This IV opioid process persists as the standard of care for patients with clinically apparent long-bone extremity fractures in our PED, despite numerous changes in the clinical microenvironment (Table 1).

**Table 1.**
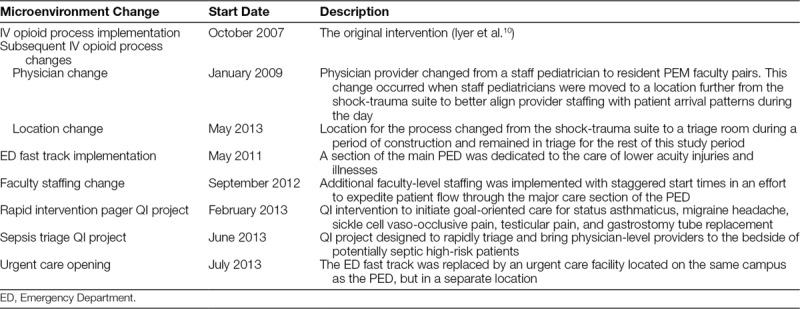
Changes in the Clinical Microenvironment

### Study Objectives

The objective of this study was to explore the long-term sustainability and unintended consequences of a QI intervention within a dynamic PED. This study had 2 specific aims. First, we explored the impact of microenvironment changes on the sustainability of successful QI work to improve the timeliness of IV opioids for patients with long-bone extremity fractures. Identifying and quantifying the effects of system variation may inform sustainability plans by helping QI teams better understand the impact of these changes on existing processes and outcomes. We hypothesized that the proportion of patients receiving IV opioids in less than 45 minutes had not changed since 2007, despite alterations to the clinical microenvironment.

Second, we sought to explore hypothesized unintended consequences of this QI implementation. Unintended consequences are defined here as consequences of the QI work not foreseen and not accounted for by the balancing measures of the original improvement team. By facilitating IV catheter placement, we hypothesized providers may be biased toward IV pain medications when other routes (e.g., oral, intranasal) may be equally appropriate. This is an important consideration because IV catheter placement in children is a potentially stressful, painful, and resource-intensive procedure.^12,13^ We hypothesized that the proportion of patients with long-bone extremity fractures receiving IV access for a single dose of IV opioid, but not requiring procedural sedation, had increased. Additionally, we hypothesized that the population of patients to whom the IV opioid process is applied has broadened to include those with other painful injuries besides long-bone extremity fractures. This is important because the IV opioid process removes providers from their primary assignment, interrupts care of other patients, and may result in undue process burden.

## METHODS

This was a retrospective observational study using data from the electronic medical record (EMR) for patient visits from January 1, 2007, to June 30, 2014. The setting for this study was a large, urban, tertiary, academic PED and level 1 pediatric trauma center with approximately 66,000 annual visits. The PED is staffed by fellowship-trained pediatric emergency medicine (PEM) faculty and resident trainees at all times. Board-certified/eligible pediatricians and nurse practitioners provide additional staffing during busy times and often practice independently for low-acuity patients. The study was conducted with approval by the institutional review board of Cincinnati Children’s Hospital Medical Center.

### Study Population

Patients were included if diagnosed with a long-bone extremity fracture, specified by the International Classification of Diseases (ICD-9) code prefixes 812, 813, 820, 821, 823, and 824. Patients were excluded if they presented with critical illness as indicated by receipt of resuscitation medications or paralytics. To determine if the population of patients receiving the IV opioid process included those without long-bone extremity fractures, we included all patients who had an IV opioid process triage designation in the EMR.

### Measures

#### Timely IV Opioids

We calculated the monthly proportion of patients who received timely IV opioids. The denominator was the number of patients with long-bone extremity fractures who received at least 1 dose of IV opioid. The numerator was the number of patients who received their first dose of IV opioid within 45 minutes of arrival in the PED, matching the definition of Iyer et al.^10^

#### Unintended Consequences

We measured the monthly proportion of patients who experienced potentially avoidable IV catheter placement, which was defined as placement for a single dose of IV opioid with no other medication administered through the IV catheter. The denominator was the number of patients with a long-bone extremity fracture who received at least 1 dose of IV opioid. The numerator was the number of patients who received only 1 dose of IV opioid and no other intravenous medications.

Finally, we examined the proportion of patients who were triaged to the IV opioid process but were not diagnosed with a long-bone extremity fracture. Diagnosis codes were collected and summarized for all patients with a triage designation for the IV opioid process from November 2012 to June 2014, which is when this triage designation became available in the EMR.

### Analysis

Annotated monthly p-charts were used to display the proportion of patients who received timely IV opioids and the proportion who received potentially avoidable IV catheterization. Data leading up to implementation of the initial IV opioid process was used to calculate the baseline centerline and control limits. Significant shifts in the measures (i.e., special cause variation) were identified using traditionally accepted rules for statistical process control.^14^

Changes in the clinical microenvironment of the PED were hypothesized to impact the success of the primary outcome. These microenvironment changes are described in Table 1 and include modifications to the original IV opioid process, new QI implementations, and alterations in staffing intended to optimize efficiency. These changes were identified by the study authors, physicians engaged in QI, and leadership within the division who were knowledgeable about system level changes during this study period. Time series models were developed to determine which, if any, of these changes were associated with shifts in the proportion of patients receiving IV opioids within 45 minutes.^15^ First, we developed a time series model for each individual microenvironment change that occurred after implementation of the IV opioid process. The dependent variable in each model was the monthly proportion of patients receiving an IV opioid within 45 minutes. The independent variables were (1) an indicator variable denoting the microenvironment change, (2) a variable to account for temporal trends for which the months were coded zero up to the change and numbered consecutively thereafter, (3) an overall secular trend variable for which months were numbered consecutively, (4) an indicator variable to control for the initial IV opioid process implementation, and (5) the monthly proportion of patients who received intranasal fentanyl delivered via a nasal spray. We controlled for intranasal fentanyl because a patient’s time to first IV opioid may be delayed if their pain was adequately controlled using the intranasal route, which has shown equivalent efficacy to IV opioids in children with fractures.^16^ Thus, we clinically did not consider administration of intranasal fentanyl a failure of the IV opioid process. Intranasal fentanyl was first used in this study population in 2009 and increased to 8% of patients by 2014 (data not shown). We included both a linear and quadratic term for intranasal fentanyl in the time series models because the monthly proportion of patients who received intranasal fentanyl exhibited a slight curvilinear pattern. A final time series model was developed by including all microenvironment changes that were significant, controlling for the implementation of the initial IV opioid process and the use of intranasal fentanyl. SAS PROC AUTOREG (SAS v9.3, SAS Institute, Inc., Cary, N.C.) was used to analyze the data to account for autocorrelation.

## RESULTS

### Sustainability of Timely IV Opioids

**Fig. 2.**
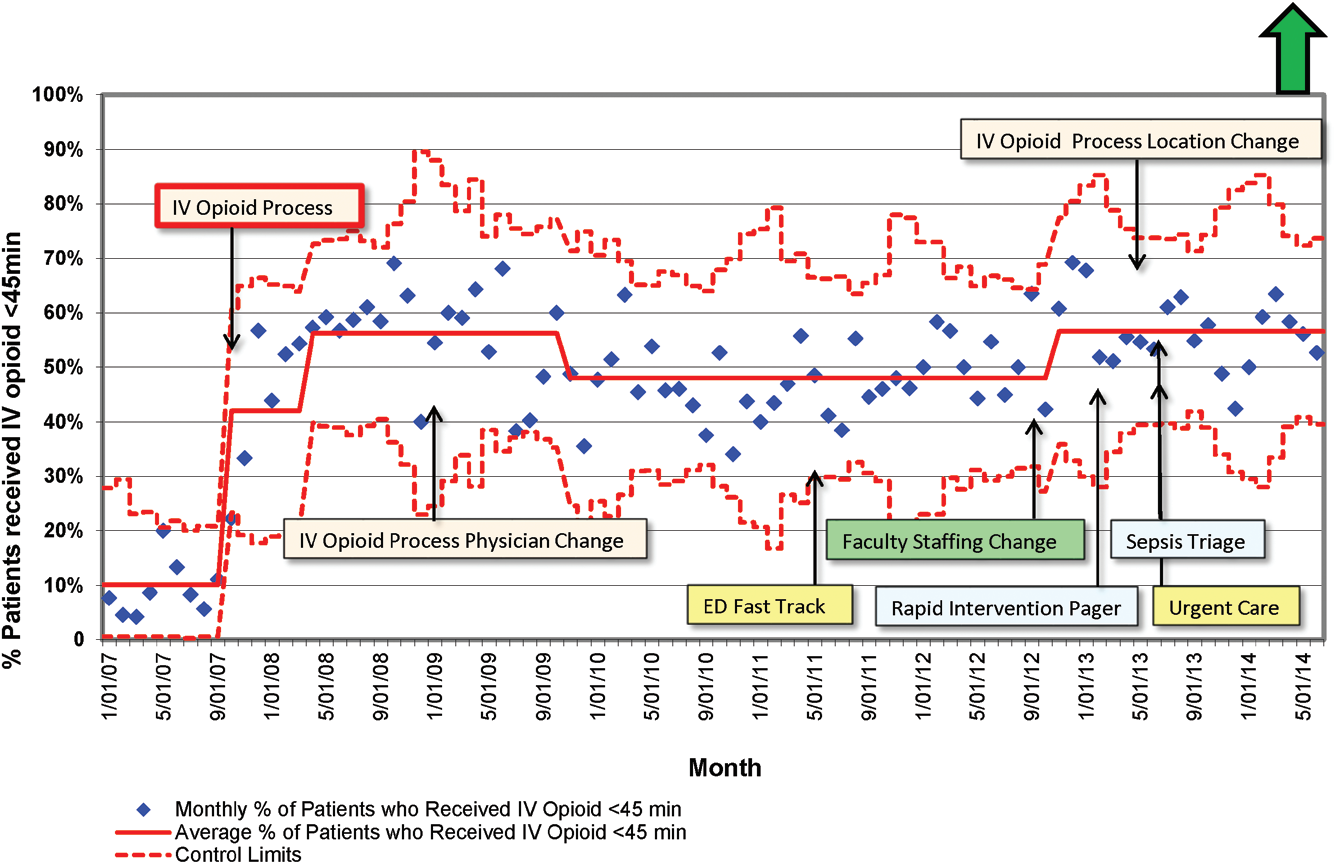
Proportion of patients with long-bone extremity fractures who received their first dose of IV opioid within 45 minutes of arrival (p-chart).

Figure 2 is a p-chart that depicts the variation in the primary outcome. The center line was recalculated when special cause variation was indicated. The annotations indicate when the implementation of the initial IV opioid process occurred followed by each microenvironment change. Overall, it shows that the gains of the original improvement team were sustained. There were, however, 2 instances of special cause variation during the postimplementation period: a negative shift beginning in September 2009 followed by a positive shift in November 2012.

Table 2 summarizes the results of the time series models for each microenvironment change. It shows that the microenvironment change in which the provider for the IV opioid process changed from a staff pediatrician to a resident PEM faculty pair was significant (*P* = 0.01) after controlling for the IV opioid process implementation and intranasal fentanyl use. Also, the altered distribution of faculty staffing hours was significant (*P* = 0.02).

**Table 2.**
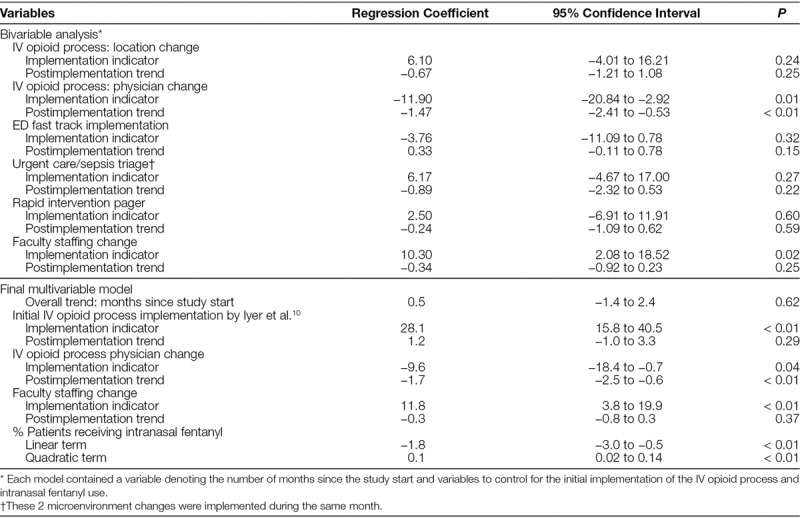
Results from the bivariable and multivariable time Series Analyses Describing the Association of Microenvironment Changes with the Proportion of Patients Receiving IV Opioids within 45 Minutes

Table 2 also summarizes the results of the final time series model. The original implementation of the IV opioid process and adjusting the distribution of faculty staffing hours were associated with an increase in the proportion of patients receiving timely opioids of 28% and 12%, respectively. Switching the type of physician who responded to the IV opioid process from a staff pediatrician to a resident PEM faculty pair was associated with a decrease of 10%. The use of intranasal fentanyl had a small negative association with the proportion of patients receiving timely IV opioids. With 1 exception, these results align with the p-chart in Figure 2. Although the type physician who responded in the IV opioid process was changed in January 2009, the control chart did not indicate special cause variation until October 2009.

### Unintended Consequences

**Fig. 3.**
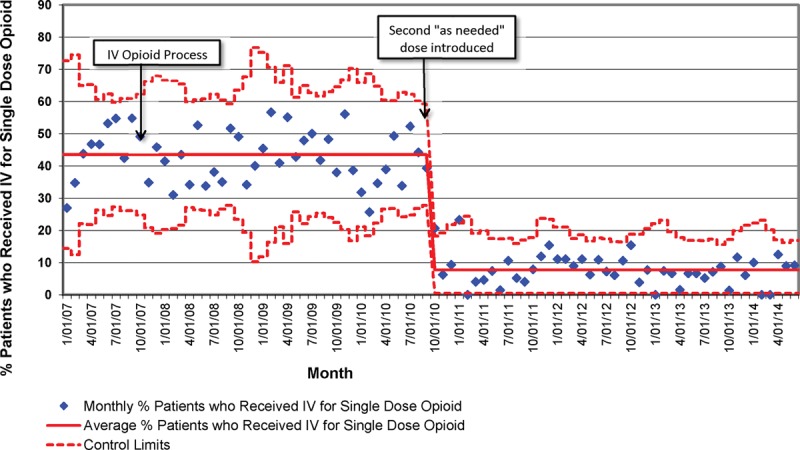
Proportion of patients with long-bone extremity fractures who received IV access for 1 dose of opioid (p-chart).

Figure 3 shows that the introduction of the IV opioid process in 2007 was not associated with an increased proportion of patients receiving an IV for a single dose of IV opioid. The large negative shift beginning in September 2010 coincided with allowing nurses to administer a second “as needed” dose of IV morphine for patients with moderate-to-severe pain as assessed on an age-appropriate, validated pain assessment tool. From November 2012 to June 2014, 944 patients were designated in the EMR as having received the IV opioid process. Of those 944 patients, 22% did not have a long-bone extremity fracture diagnosis. Table 3 shows the diagnoses attributed to those without long-bone extremity fractures. Only 1% of those without a fracture diagnosis had a noninjury, nonpain-related diagnosis code.

**Table 3.**
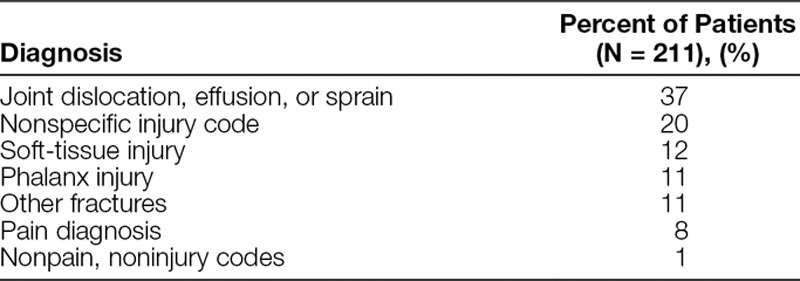
Diagnoses of Patients without Acute Long-Bone Extremity Fractures Who Were Triaged to the IV Opioid Process

## DISCUSSION

The proportion of patients receiving timely IV opioids remained above the preimplementation baseline despite numerous changes in the clinical microenvironment. The introduction of a resident physician increased the time between patient arrival and IV opioid administration, which was later mitigated when the faculty staffing model was adjusted to optimize the number of faculty and their availability for bedside care during the busier times of the day. These results are shown in both our final time series analysis model and the p-chart in Figure 2. These results suggest that for an intervention requiring rapid decision making and action, the type of provider responding may be an important variable to consider when designing the process.

Broad categories of factors contributing to sustainability include context, resources, funding, culture, and adaptability. No further improvement personnel or financial resources were dedicated to the IV opioid process beyond the initial improvement project, which ended 18 months after implementation. The influence of culture and adaptability are harder to quantify but are still important considerations in the reporting of QI work.^17^ This IV opioid process was developed in an institution with substantial support for QI, fostering a culture of change and adaptability. Methods to measure and quantitatively report the impact of institutional and leadership support, however, are still not well described.^17^

A systematic review identified numerous gaps in the healthcare literature, which limit our understanding of sustained improvement outcomes.^17^ One particular gap includes the lack of a standard definition of sustainability.^18^ Enhanced sustainability, however, has been described as incorporating a process into an organization’s operations such that process outcomes are mitigated over time with changes in the microenvironment (Fig. 2).^18^ Sustainability also depends on the level of reliability of the QI interventions. High reliability interventions are more likely to consistently produce intended outcomes, despite inconsistencies in human behavior.^19^ Although processes with lower levels of reliability may be initially successful (e.g., checklists or education modules), the outcomes can be difficult to sustain.^20^ Sustainability requires attention to process design and incorporation of the process into the operations of the global system.^20^ The IV opioid process was designed as a level of reliability 10^–2^ intervention as it took advantage of the existing behavior of emergently responding to pages (Fig. 1). Furthermore, the IV opioid process mimicked our existing trauma and medical resuscitation alert systems, which were already ingrained in the workflow of our PED.

Improvement methodologies suggest sustainability should be measured as persistence of the process without variation.^20^ A successful and long-standing process, however, needs to accommodate advances in healthcare while maintaining its core structure to avoid entrenchment in outdated medical practices.^17^ The strength of the IV opioid process is the persistence of its basic structure to identify patients with apparent painful injuries and rapidly gather a team to provide pain medication. Even though a sufficient team was gathered for IV catheter placement, the exact means of analgesia was not prescribed or mandated by the process. Bedside providers maintained the freedom to tailor pain medication orders to the needs of the patient. This specific feature of the IV opioid process allowed for intranasal fentanyl use in the years following implementation without changing the core structure of the process.

Because the IV opioid process was directed at expediting IV pain medication, we speculated this may bias providers toward placing IV catheters when alternative routes may be appropriate. This study shows that the number of IVs placed for only 1 dose of IV opioid medication did not increase among patients with long-bone extremity fractures. In fact, providers capitalized on the IV opioid process as a point in time to place additional “as needed” medication orders and increase the provision of opioid medications to adequately control acute pain during PED visits. As seen in Figure 3, substantially fewer patients received only 1 dose of IV opioid medication after the “as needed” orders were implemented.

We also hypothesized that the IV opioid process was being used for patients without clinically apparent long-bone extremity fractures, potentially causing undue process burden on our PED system. We found that almost all patients subject to the IV opioid process had painful injuries warranting timely analgesia including the 22% of patients without long-bone extremity fracture diagnoses. This reflects a potentially positive unintended consequence with spread of a successful process to other groups to expedite pain management for a larger segment of patients.

This study highlights several avenues for continued improvement work. First, our current outcome measure (percentage of patients receiving timely IV opioid) does not provide enough granular information about sources of variability in time to opioid. Future work should examine mean time to IV opioid using X-bar and S charts segmented by provider and patient characteristics to better understand variation and develop targeted interventions. Second, as the system moves toward increased use of intranasal pain medications, it will be important to refine the current process to adapt to this change and continue to decrease the time to analgesia. It is also important to characterize the impact of intranasal medications on reducing the burden of IV catheterization in children with fractures. Lastly, future studies on opioid use in the emergency department will need to balance the importance of adequate analgesia in the midst of a national opioid abuse crisis.

### Limitations

Because this study was conducted retrospectively using EMR data, we used diagnosis codes to identify eligible patients. As a result, we could not discern whether patients we included had a “clinically apparent” long-bone extremity fracture, the population of patients to whom the initial IV opioid process was intended. Although inclusion of children without clinically apparent fractures could bias our results, we are confident this bias was consistent during the study period. Also, like most single-center publications of QI projects, the work done by the initial improvement team and sustainability of its outcomes may not be readily generalizable. For example, we recognize our physician staffing model may not be present or feasible at other institutions. Similar success, however, may be achieved by using physician-extending providers (e.g., advanced practice nurses, physician assistants) or restructuring provider-patient ratios to optimize the providers’ capacities. For this reason, we provided a description of each microenvironment change (Table 1) so that organizations can consider contextual factors when interpreting our results. Lastly, we recognize other microenvironment changes may exist that were not accounted for in planning this study.

## CONCLUSIONS

This study is a first step in understanding long-term sustainability of our QI work. Specifically, we found our initial improvement gains were robust despite multiple changes in the clinical microenvironment and that optimization of physician staffing and physician responsibilities in a PED may be especially important to consider when planning future improvement initiatives. Our findings support the importance of higher reliability interventions, such as identification and utilization of existing patterns of behavior as being particularly high-yield for improvement teams striving to achieve sustained outcomes. Future research should focus on the long-term sustainability of outcomes resulting from improvement work taking into account the organizational characteristics and processes that both enhance and inhibit sustainability, such as those outlined by the Institute for Healthcare Improvement.^21^ Understanding sustainability and its drivers is key to transforming the quality of care we provide to patients and families.

## ACKNOWLEDGMENTS

Thank you to Richard Ruddy, MD, for mentorship and support in planning this study. Thank you also to the original improvement team members: Srikant Iyer, MD, Charles Schubert, MD, Scott Reeves, MD, and Jenny Oehler, BSN.

## DISCLOSURE

The authors have no financial interest to declare in relation to the content of this article.
